# Human Stromal (Mesenchymal) Stem Cells from Bone Marrow, Adipose Tissue and Skin Exhibit Differences in Molecular Phenotype and Differentiation Potential

**DOI:** 10.1007/s12015-012-9365-8

**Published:** 2012-04-14

**Authors:** May Al-Nbaheen, Radhakrishnan vishnubalaji, Dalia Ali, Amel Bouslimi, Fawzi Al-Jassir, Matthias Megges, Alessandro Prigione, James Adjaye, Moustapha Kassem, Abdullah Aldahmash

**Affiliations:** 1 Stem Cell Unit, Department of Anatomy 28, College of Medicine, King Saud University, P.O. Box 2925, Riyadh, 11461 Kingdom of Saudi Arabia; 2 Department of Orthopedic Surgery, King Khalid University Hospital, College of Medicine, King Saud University, Riyadh, Kingdom of Saudi Arabia; 3 Department of Vertebrate Genomics, Molecular Embryology and Aging group, Max Planck Institute for Molecular Genetics, Ihnestr. 63-73, 14195 Berlin, Germany; 4 Endocrine Research Laboratory (KMEB), Department of Endocrinology and Metabolism, Odense University Hospital & University of Southern Denmark, Odense, Denmark

**Keywords:** Stromal cells, Mesenchymal stem cell, Adipose tissue, Bone marrow, Skin, DNA microarray

## Abstract

Human stromal (mesenchymal) stem cells (hMSCs) are multipotent stem cells with ability to differentiate into mesoderm-type cells e.g. osteoblasts and adipocytes and thus they are being introduced into clinical trials for tissue regeneration. Traditionally, hMSCs have been isolated from bone marrow, but the number of cells obtained is limited. Here, we compared the MSC-like cell populations, obtained from alternative sources for MSC: adipose tissue and skin, with the standard phenotype of human bone marrow MSC (BM-MSCs). MSC from human adipose tissue (human adipose stromal cells (hATSCs)) and human skin (human adult skin stromal cells, (hASSCs) and human new-born skin stromal cells (hNSSCs)) grew readily in culture and the growth rate was highest in hNSSCs and lowest in hATSCs. Compared with phenotype of hBM-MSC, all cell populations were CD34^−^, CD45^−^, CD14^−^, CD31^−^, HLA-DR^−^, CD13^+^, CD29^+^, CD44^+^, CD73^+^, CD90^+^,and CD105^+^. When exposed to in vitro differentiation, hATSCs, hASSCs and hNSSCs exhibited quantitative differences in their ability to differentiate into adipocytes and to osteoblastic cells. Using a microarray-based approach we have unveiled a common MSC molecular signature composed of 33 CD markers including known MSC markers and several novel markers e.g. CD165, CD276, and CD82. However, significant differences in the molecular phenotype between these different stromal cell populations were observed suggesting ontological and functional differences. In conclusion, MSC populations obtained from different tissues exhibit significant differences in their proliferation, differentiation and molecular phenotype, which should be taken into consideration when planning their use in clinical protocols.

**Electronic supplementary material:**

The online version of this article (doi:10.1007/s12015-012-9365-8) contains supplementary material, which is available to authorized users.

## Introduction

Human stromal stem cells (also known as mesenchymal stem cells or multipotent stromal stem cells) (hMSC) are a group of clonogenic cells capable of self-renewal and multilineage differentiation into mesoderm-type cells e.g. osteoblasts, adipocytes and chondrocytes [1, 2]. MSC are being introduced in a number of clinical trials for tissue repair e.g. bone and cartilage defects, for the enhancement of tissue regeneration e.g. myocardial infarction, and immune modulation e.g. graft-versus-host disease (GvHD) [1, 3]. The initial results from these trials are very encouraging. The standard site for obtaining human MSC is bone marrow where the cells are located on the abluminal surface of blood vessels [4]. However, one limitation for obtaining hMSC from bone marrow is the difficulty of obtaining enough number of cells required for clinical studies [5]. During the recent years, MSC-like populations have been obtained from a wide range of tissues e.g. adipose tissue [6], skin [7], blood [8], umblical cord blood [9], teeth [10], pancreas [11] and liver [12]. Among all these tissues, adipose tissue and skin are attractive choices to obtain cells needed for clinical studies due to the ease of obtaining clinical samples.

Adipose tissue used for providing MSC is usually obtained during operative procedure e.g. liposuction [13] and human adipose tissue derived stromal cells (hATSCs) have been reported to exhibit a similar phenotype to that of human bone marrow MSC (hBM-MSCs) [6, 13] . Also recently, it has been reported that stromal cultures of foreskin and skin can generate MSC-like cells with differentiation capacity into mesodermal cells (adipocytes, osteoblasts, chondrocytes) and possibly to cells from the ectodermal cells and endodermal lineages in vitro [14–16]. However, similarities and differences of these different cell populations are not clearly defined.

The aim of the present study was to compare stromal cell populations obtained from two clinically relevant sources: adipose tissue and skin with the standard bone marrow-derived MSC. In addition, we employed microarray-based gene expression profiling in order to compare the molecular phenotype of these cell populations.

## Material and Methods

### Cell Culture

We obtained samples of adipose tissue and dermal skin from patients undergoing abdominal bariatric surgery, lipectomy, knee replacement or gastrointestinal operations. Fresh foreskin specimens were obtained from 2–3 day old male babies. None of the patients had malignant disease and all provided written informed consent. The project was approved by the Institution Review Board of King Saud University Medical College and Hospital (10-2815-IRB).

Unless otherwise stated, the basal culture medium used in all experiments is Dulbecco’s Modified Eagle Medium (DMEM) (supplemented with D-glucose 4500 mg/L, 4 mM L-Glutamine and 110 mg/L Sodium Pyruvate, 10 %), Fetal Bovine Serum (FBS) (10 %), Penicillin-Streptomycin(P/S) (1 %) and non-essential amino acids (1 %). All reagents were purchased from Gibco-Invitrogen, USA.

### Human Adipose-Tissue Stromal Cells (hATSCs)

The adipose tissues were washed 3 to 4 times using Phosphate buffered saline (PBS), minced and incubated in 1 % collagenase type 1 for 45 min at 37 °C. Mature adipocytes and undigested tissue fragments were separated from pellets of stromal vascular fraction (SVF) by centrifugation at 500 g for 15 min. SVF cells were re-suspended in culture medium and plated in 25 cm^2^ tissue culture flasks and maintained in a humidified incubator at 37 °C and 5 % CO_2_. All non-adherent cells were removed after 24 h. Cells were fed with new medium for every 3–4 days until 70–80 % confluence. For all experiments cells were used at passage 4 with division ratio 1:3.

### Human Skin Stromal Cells

Skin stromal cells were derived from two sources: foreskin samples (human new-born skin stromal cells, hNSSCs) and from abdominal or knee skin samples (human adult skin stromal cells, hASSCs). The skin specimens were washed in PBS and the subcutaneous tissues (hypodermis) were mechanically dissected and removed. The samples were cut into small pieces ≈3 mm and employed as an explant culture with the dermis layer lying on the culture surface. The tissues were maintained in a humidified incubator at 37 °C and 5 % CO_2._ For all experiments cells were used at passage 4 with division ratio 1:3. For DNA microarray studies, two commercially available non-stem fibroblastic cell lines were included: neonatal foreskin fibroblasts HFF1(ATCC # SCRC-1041) and BJ (ATCC # SCRC-2522).

### Human Bone Marrow-Derived MSC

As a model for human bone marrow derived MSC (hBM-MSCs), we employed a well characterized hMSC cell line that has been telomerized by the human telomerase reversetranscriptase gene (hTERT) transduction and known as hMSC-TERT [17]. The hMSC-TERT express all known markers and similar differentiation capacity of normal hBM-MSCs in vitro and in vivo [18]. For the DNA microarray studies, we included as a control, primary bone marrow derived MSC that were obtained from haematologically normal, osteoarthritic donor patients undergoing routine total hip-replacement surgery using STRO-1 antibody by immune magnetic panning (Kindly provided by Dr Emmajayne Kingham and Professor Richard Oreffo, University of Southampton, UK) .

### Cell Proliferation

Proliferation rates of hATSCs, hASSCs and hNSSCs were determined by counting cell number and calculating population doubling (PD) rate. The cells were cultured in 6 cm^2^ tissue culture petri dish at cell density 8000 cells/cm^2^. At confluency, the cells were trypsinized and counted manually by hemocytometer. At each passage, population doubling was determined by the formula: logN/log2 where N is the number of cells at confluence divided by the initial cell number. Cumulative PD level is the sum of population doublings and PD rate is PD/time in culture.

### Colony Forming Unit-Fibroblast (CFU-F) Assay

hATSCs, hASSCs and hNSSCs were plated at 10^3^ cells in 6-cm petri dishes and allowed to grow for 15 days. The cultures were terminated and stained with crystal violet for colony visualization. A colony was defined as a group of cells (>40). The colonies were counted manually under an inverted microscope.

### Cell Differentiation

#### Osteoblast Differentiation

Cells were cultured in basal medium till 70–80 % confluence. Osteogenic induction medium composed of DMEM containing 10 % FBS, 1 % P/S, 50 μg/mL L-ascorbic acid (Wako Chemicals GmbH, Neuss, Germany), 10 mM β-glycerophosphate (Sigma), and 10 nM calcitriol[(1α,25-dihydroxy vitamin D3) (sigma)], 10 nM dexamethasone (Sigma) was added and was changed every 3 days. Control cultures were maintained in vehicle-containing basal medium.

#### Adipocyte Differentiation

Cells were cultured in basal medium until 90–100 % confluence and then transferred to DMEM medium containing adipogenic-induction mixture containing 10 % FBS, 10 % Horse Serum (Sigma), 1 % P/S, 100 nM dexamethasone, 0.45 mM isobutyl methyl xanthine [(IBMX) (Sigma)], 3 μg/mL insulin (Sigma), and 1 μM Rosiglitazone [(BRL49653) (Novo Nordisk, Bagsvaerd, Denmark)]. The adipogenic induction medium was replaced every 3 days. Control cells were cultured in vehicle-containing basal medium.

### Cytochemical Assays

#### Alkaline Phosphatase (ALP) Staining for Osteoblasts

Cells were washed in PBS, fixed in acetone/citrate buffer and incubated with ALP substrate solution (naphthol AS-TR phosphate 0.1M Tris buffer, pH 9.0) for 1 h at room temperature.

#### Oil Red-O Staining for Adipocytes

Cells were washed in PBS, fixed in 4 % formaldehyde and stained for 1 h at room temperature with filtered Oil red-O staining solution (prepared by dissolving 0.5 g Oil red-O powder in 60 % isopropanol).

### Immunofluorescence Staining

Cells were fixed with 4 % cold paraformaldehyde (Sigma) for 15 min and permeabilized with 0.1 % Triton X-100 (Sigma) for 10 min. After washing with PBS, cells were treated with 3 % bovine serum albumin (BSA, Sigma) for 30 min, followed by incubation with primary antibody (purified mouse anti-vimentin, BD Pharminogen) diluted in PBS (1:100) at 4 °C overnight. After removal of primary antibodies, cells were washed three times with PBS, and the secondary antibody (Goat polyclonal to anti mouse IgG, Abcam) conjugated to FITC was added (1:4000) and incubated for 1 h at room temperature. Cells were washed three times with PBS, and mounted with a medium containing DAPI to detect nuclei (VectaShield; Vector Labs, Burlingame, CA).

### Flow Cytometry (FACS) Analysis

Cells were harvested by use of 0.05 % trypsin-EDTA for 5 min at 37 °C, recovered by centrifugation at 200 g for 5 min, washed twice in ice-cold PBS supplemented with 2 % FBS and re-suspended at a concentration of 10^5^ cells/antibody test. Ten μL of PE-conjugated mouse anti-human CD146, CD73, CD29 and HLA-DR, FITC-conjugated mouse anti-human CD34, CD90, CD45, CD13 and CD31, APC-conjugated mouse anti-human CD105, CD14 and CD44 antibodies (all from BD Biosciences, except that the monoclonal antibody against human CD105, was from R&D systems) were used. Negative control staining was performed using a FITC/PE/APC-conjugated mouse IgG1 isotype antibodies. After storage for 30 min at room temperature in the dark, cells were washed with PBS, re-suspended in 500 μL of PBS and analyzed in the BD FACS Calibur flow cytometer (BD Biosciences). Living cells were gated in a dot plot of forward versus side scatter signals acquired on linear scale. At least, 8000 gated events were acquired on a log fluorescence scale. Positive staining was distinct as the emission of a fluorescence signal that surpassed levels achieved by >99 % of control cell population stained with corresponding isotype antibodies. The ratios of fluorescence signals versus scatter signals were calculated and histograms were generated using the software Cell Quest Pro Software Version 3.3 (BD Biosciences).

### Reverse Transcriptase (RT)-Real-Time Quantitative Polymerase Chain Reaction (qPCR)

Total RNA was extracted using MagNA pure compact RNA isolation kit (Roche Applied Science, Germany. Cat No: 04802993001) in automated MagNA pure compact system (Roche, Germany). cDNA synthesis and Polymerase chain reaction (PCR) samples were prepared using a iScript One-step RT-PCR Kit with SYBER Green (Bio-Rad, USA) and run on a Light Cycler (Roche) PCR machine. Relative quantification of PCR products were based on value differences between the target and β-actin control using the 2^−ΔΔCT^ method., The following RT-PCR primers (all from Invitrogen limited, UK) were used to detect the expression of specific ß-actin (forward: TGTGCCCATCTACGAGGGGTATGC, reverse: GGTACATGGTGGTGCCGCCAGACA, amplify 448 bp), ALP (forward: ACGTGGCTAAGAATGTCATC, reverse: CTGGTAGGCGATGTCCTTA, amplify 475 bp), Osteocalcin (forward: AGAGCGACACCCTAGAC, reverse: CATGAGAGCCCTCACA, amplify 310 bp), Osteopontin (forward: GGTGATGTCCTCGTCTGTA, reverse: CCAAGTAAGTCCAACGAAAG, amplify 347 bp) PPAR-*γ* 2 (forward: CTCCACTTTGATTGCACTTTGG, reverse: TTCTCCTAT TGACCCAGAAAGC, amplify 307 bp), aP2 (forward: TGGTTGATTTTCCATCCCAT, reverse: GCCAGGAATTTGACGAAGTC, amplify 107 bp), Adiponectin (forward: ATGTCTCCCTTAGGACCAATAAG, reverse: TGTTGCTGGGAGCTGTTCTACTG, amplify 234 bp. The relative abundance of target mRNA was expressed relative to β-actin gene expression.

### Microarray-Based Global Gene Expression Analysis

Total RNA was isolated using the GeneMatrix Universal RNA Purification Kit (Cat. E 3598-02, Roboklon, Berlin, Germany) and quality-checked by Nanodrop analysis (Nanodrop Technologies, Wilmington, DE, USA). 400 ng of total RNA was used as input for generating biotin-labeled cRNA (Ambion, Austin, TX, United States). cRNA samples were then hybridized onto Illumina human-8 BeadChips version 3. Hybridizations, washing, Cy3-streptavidin staining and scanning were performed on the Illumina BeadStation 500 platform (Illumina, San Diego, CA, USA), according to the manufacturer’s instruction. hMSC-TERT was hybridized in duplicates, while triplicates were used for the following samples: hNSSCs, hASSCs, hATSCs. Expression data analysis was carried out using the BeadStudio software 3.0 (Illumina, San Diego, CA, USA). Raw data were background-subtracted, normalized using the “rank invariant” algorithm, and filtered for significant expression on the basis of negative control beads. Genes were considered significantly expressed with detection p values ≤ 0.01. Differential expression analysis was performed with the illumina custom method using hMSC-TERT as reference control. The following parameters were set to identify statistical significance: differential p values ≤ 0.01, fold change ratio >1.5. Pathway analysis was performed using DAVID Bioinformatics Resources 6.7 (http://david.abcc.ncifcrf.gov). Heatmap picture was generated using Microarray Software Suite TM4 (TM4.org).

### Statistical Analysis

All results are based on at least 3 independent experiments and are expressed as mean % ± SD for 6 donors in each group. The One-Way ANOVA was used to analyze results of FACS. Post-hoc testing was performed for intergroup comparison using student *T*-test. Student *t* test was used to compare the mean values of PD rates between groups. Value of *P <* 0.05 was considered statistically significant. The SPSS software package (version 17.0; SPSS Inc., USA) was used for the statistical testing.

## Results

### Cell Morphology

**Fig. 1:**
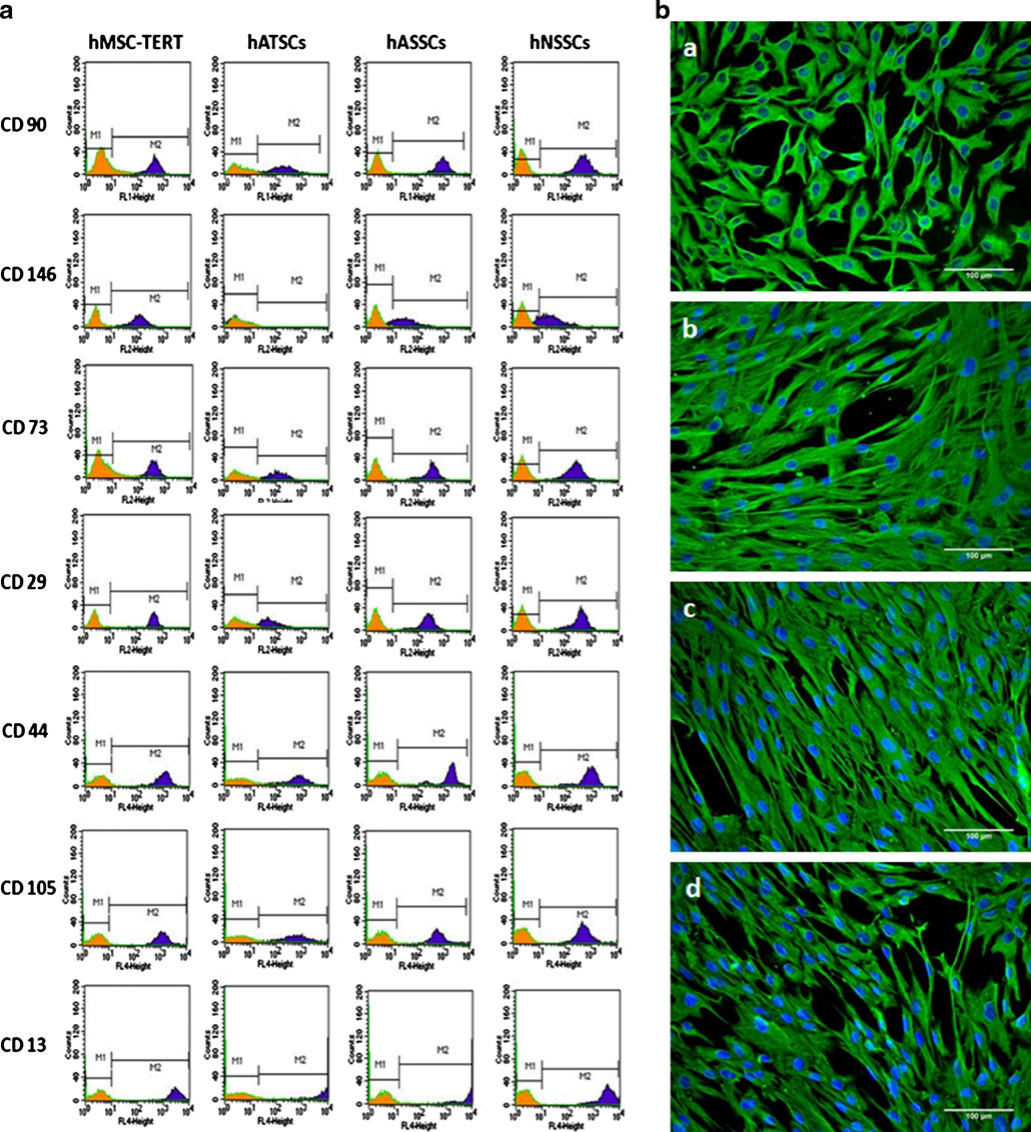
Phenotypic analysis hMSC-TERT, hATSCs, hASSCs and hNSSCs. The human bone marrow stromal (mesenchymal) stem cell (hMSC) immortalized with human telomerase reverse transcriptase gene (hMSC-TERT) and stromal cells derived from adipose tissue (hATSCs), adult dermal skin (hASSCs) and neonatal foreskin (hNSSCs) cells were cultured using plastic adherence. **a** Flow cytometry analysis of CD cell surface proteins. Filled histograms represent cells stained with the corresponding isotype control antibody. Five thousand events were collected and analyzed. **b** Immunofluorescence based detection of Vimentin expression and visualization of nuclei using DAPI

hATSCs, hASSCs and hNSSCs as well as hMSC-TERT exhibited fibroblast-like appearance with no distinct morphological differences (Supplementary Figure 1). Also, immunocytochemical staining for vimentin which a general marker for mesenchymal cells, demonstrated similar staining pattern among the four cell populations (Fig. 1b).

### FACS Analysis for Surface Marker Expression

hATSCs, hASSCs and hNSSCs and hMSC-TERT were analyzed for expression of CD markers known to be expressed by MSC (Fig. 1a). All the cell populations were negative for the hematopoietic and endothelial lineage markers CD34, CD45, CD14, CD31, as well as for the MHC class II molecule: HLADR. The cell populations were positive for known hBM-MSC markers and the percentage of positive cells were similar in all four cell populations except for CD146 that was expressed at low levels (5 %) in hATSCs (Supplementary Table 1).

### Cell Proliferation

**Fig. 2:**
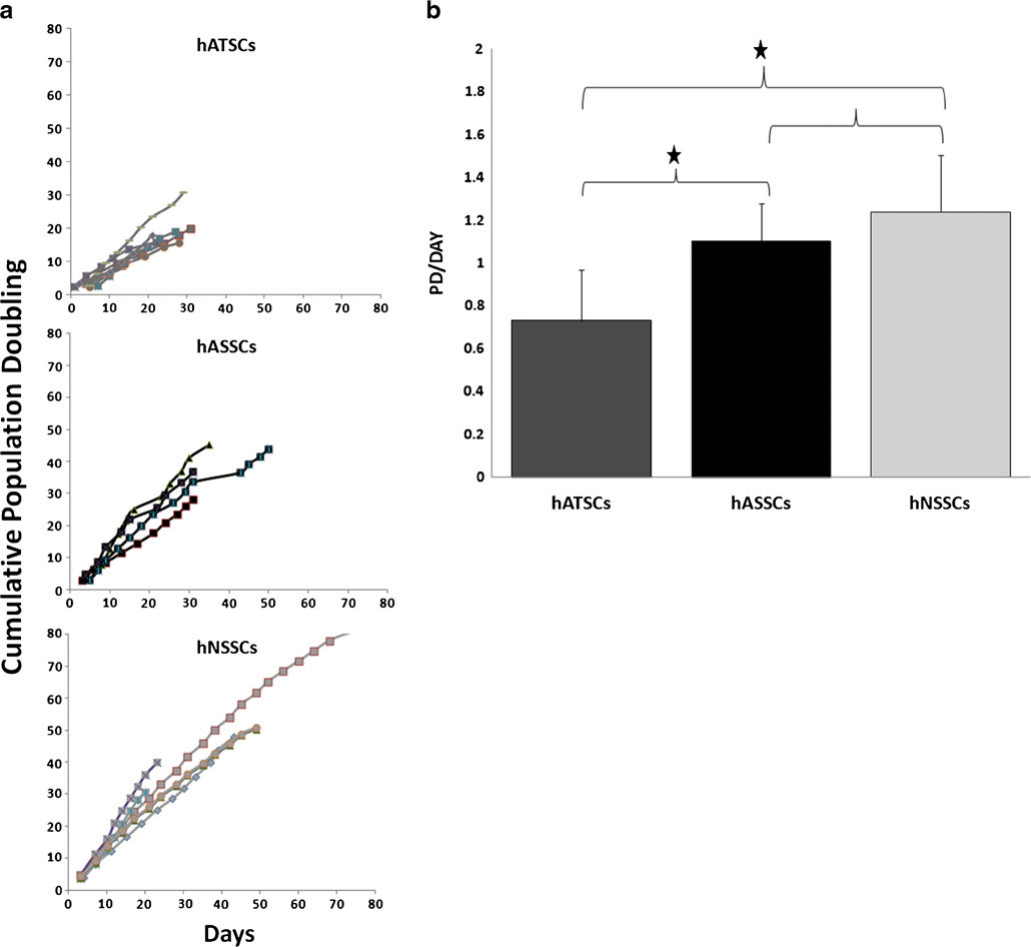
Proliferation potential of hATSCs, hASSCs and hNSSCs. Stromal cells derived from adipose tissue (hATSCs), adult dermal skin (hASSCs) and neonatal foreskin (hNSSCs) cells were cultured using plastic adherence. **a** hATSCs (*n* = 7), hASSCs (*n* = 5) and hNSSCs (*n* = 6) cumulative population doublings (PD) during long-term culture. **b** PD rate of hATSCs, hASSCs and hNSSCs. *p < 0.05

Individual growth curves of hATSCs, hASSCs and hNSSCs cell strains and the mean values of growth rate as estimated by PD/day in each cell type are presented in Figs. 2a & b. As shown in Fig. 2b, hNSSCs exhibited a higher cell proliferation rate in long-term cultures compared with hASSCs and hATSCs as evidenced by mean PD rate of 0.78, 1.13 and 1.11 PD/day, respectively.

### CFU-f Formation

hATSCs, hASSCs and hNSSCs were able to form colonies (supplementary Figure 2) and the number of colonies formed in hATSCs were lower than those formed in hASSCs and hNSSCs.

### Cell Differentiation

#### Osteoblast Differentiation

**Fig. 3:**
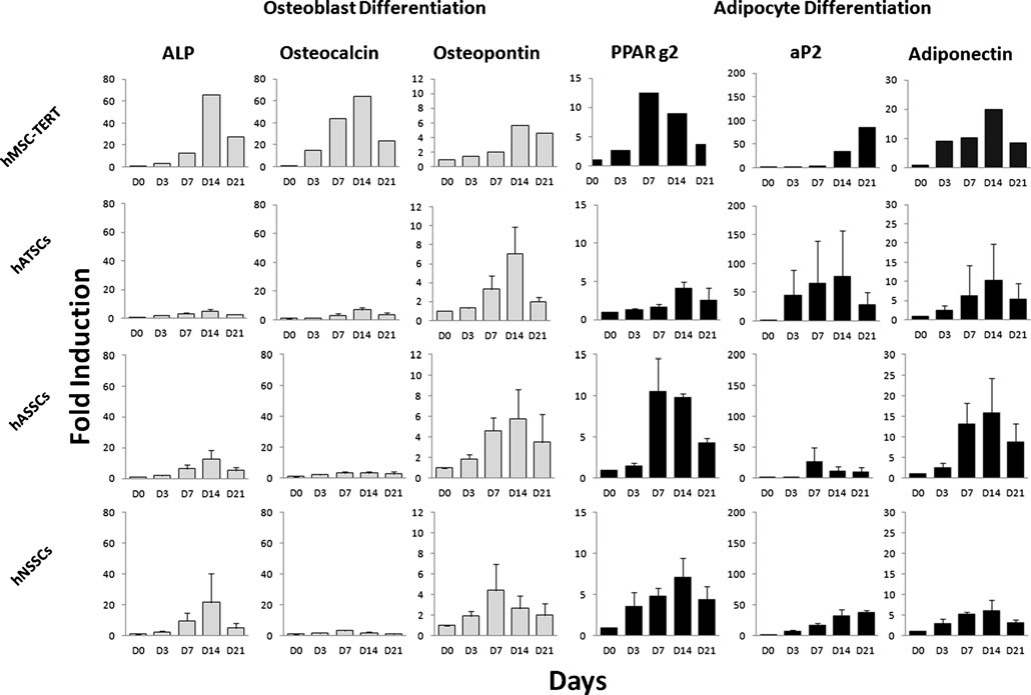
Gene expression of osteoblast and adipocyte markers during in vitro differentiation of hMSC-TERT, hATSCs, hASSCs and hNSSCs. The human bone marrow stromal (mesenchymal) stem cells (hMSC) immortalized with human telomerase reverse transcriptase gene (hMSC-TERT) and stromal cells derived from adipose tissue (hATSCs), adult dermal skin (hASSCs) and neonatal foreskin (hNSSCs) cells were cultured using plastic adherence and exposed to either osteoblast or adipocyte differentiation medium over a 21 day (D) period. Gene expression was normalized to beta-ACTIN and was represented as fold-change of non-induced D0 control cells. hMSC-TERT, hATSCs, hASSCs and hNSSCs data are shown as mean ± SD of three donor biological samples from at least two independent experiments. ALP = alkaline phosphatase, PPARg2 = Peroxisome proliferator-activated receptor gamma2, aP2 = adipocyte protein 2

hATSCs, hASSCs, hNSSCs and hMSC-TERT were exposed to 21-day in vitro osteoblast differentiation and time course expression of osteoblastic makers (ALP, osteocalcin and osteopontin) was determined (Fig. 3). Based on fold increase in expression of osteoblastic markers, induction of osteoblastic phenotype was most pronounced in hMSC-TERT. hATSCs, hNSSCs and hASSCs exhibited limited responses in expression of ALP and osteocalcin. Similar data were observed from ALP cytochemical staining where OB-induced hMSC-TERT exhibited the most intense staining followed by hATSCs and to lesser extent by hASSCs and hNSSCs (Supplementary Figure 3).

#### Adipocyte Differentiation

hATSCs, hASSCs and hNSSCs and hMSC-TERT were exposed to 21-day in vitro adipocyte differentiation and time course expression of adipocytic makers (PPARγ2, aP2 and adiponectin) was determined (Fig. 3). The four cell populations responded to adipocyte induction by up-regulation of adipocytic gene markers. Large inter-individual variation in the degree of adipogenic responses were observed among different cell strains obtained from different donors but all cell population formed lipid-filled adipcoytes. Adipocyte formation was most extensive in hATSCs (Supplementary Figure 4).

### Microarray Analysis

In order to identify the molecular phenotype of hATSCs, hASSCs, hNSSCs and hMSC-TERT cells, microarray-based gene expression was carried out. Hierarchical clustering (Supplementary Figure 5A, B) and the correlation co-efficients-R^2^ (Supplementary Figure 5C) revealed that the transcriptome of hNSSCs is much closer to that of hMSC-TERT cells (R^2^ 0.803–0.827), followed by hASSCs (R^2^ 0.774–0.832), and then hATSCs cells (R^2^ 0.641–0.791).

**Fig. 4:**
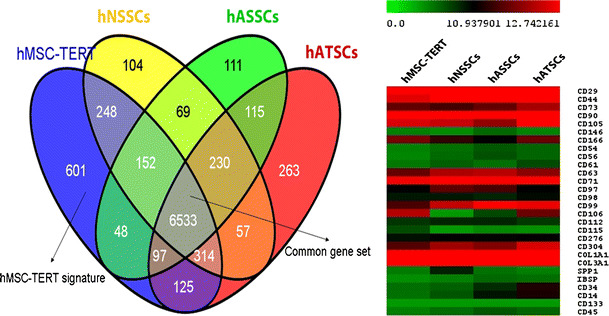
Microarray-based gene expression analysis of hMSC-TERT, hATSCs, hASSCs and hNSSCs. The human bone marrow stromal (mesenchymal) stem cells (hMSC) immortalized with human telomerase reverse transcriptase gene (hMSC-TERT) and stromal cells isolated from adipose tissue (hATSCs), adult dermal skin (hASSCs) and neonatal foreskin (hNSSCs) cells were cultured using plastic adherence. Total RNA was isolated and microarray analysis was carried out. **a** Venn diagram representing distinct and overlapping gene expression patterns between these cell populations. **b** Heat-map of a number of CD markers representing a common MSC molecular signature

To enable a clear overview of the distinct and overlapping gene expression patterns between these cell populations, a Venn diagram was constructed based on genes detected as expressed within each cell type (Fig. 4a). Full details of these groups of genes and associated pathways are presented in supplementary Table 2. A vast number of genes (*n* = 6533) are expressed in common in all the cell types, a distinct feature of this signature is the expression of known MSC surface markers such as (CD29, CD44, CD73, CD90, CD63, CD71, CD105, CD304) and the lack of expression of prototypic hematopoietic antigens such as CD34, CD11a and CD45 (Table 1). An expanded list of the expression patterns of various cell surface markers is presented in Table 1. Most notable is the core expression of 36 cell surface markers (cluster I) which we refer to as a “common MSC molecular signature”. This cluster also includes CD29, CD44, CD73, CD90, CD63, CD71, CD105, CD304. This cluster is also expressed in primary bone marrow hMSC-STRO^+^ cells. Cluster II is composed of genes of cell surface markers that are not expressed in hMSC-TERT nor in hMSC-STRO+ including CD34, CD11a and CD45. We found that 72 out of 82 surface markers (cluster I and II) reveal the same expression pattern in hMSC-TERT and hMSC-STRO^+^. Ten surface markers are expressed in hMSC-TERT, hNSSCs, hATSCs and hASSCs but not in hMSC-STRO^+^ : cluster III: surface markers CD49b, CD49d, CD115, CD117, CD164, HLA-DRA. Finally, cluster IV is composed of surface markers CD14, CD15, CD102 which are expressed in hMSC-STRO^+^, hNSSCs, hATSCs and hASSCs but not in hMSC-TERT. None of these surface markers were expressed in HFF and in BJ cell line only CD104 was expressed.

**Table 1:** A list of the expression patterns of various cell surface markers expressed in the human bone marrow stromal (mesenchymal) stem cells (hMSC) immortalized with human telomerase reverse transcriptase gene (hMSC-TERT), non-immortalized hMSCs sorted for the marker STRO-1 (hMSC-STRO^+^), stromal cells isolated from adipose tissue (hATSCs), adult dermal skin (hASSCs) and neonatal foreskin (hNSSCs) and fibroblasts cell lines HFF and BJ. Cluster I: expressed in hMSC-TERT and hMSC-STRO^+^, cluster II: not expressed in hMSC-TERT and hMSC-STRO^+^, cluster III: only expressed in hMSC-TERT, cluster IV: only expressed in hMSC-STRO^+^. + = gene expression detected, − = gene expression was absent

Gene name	Marker	hMSC-TERT	hMSC-STRO^+^	hATSCs	hNSSCs	hASSCs	HFF	BJ	Cluster
ALCAM	CD166	+	+	+	+	+	−	−	I
ANPEP	CD13	+	+	+	+	+	−	−	
B2M	B2M	+	+	+	+	+	−	−	
CD276	CD276	+	+	+	+	+	−	−	
CD44	CD44	+	+	+	+	+	−	−	
CD58	CD58	+	+	+	+	+	−	−	
CD63	CD63	+	+	+	+	+	−	−	
CD82	CD82	+	+	+	+	+	−	−	
CD97	CD97	+	+	+	+	+	−	−	
CD99	CD99	+	+	+	+	+	−	−	
COL1A1	COL1A1	+	+	+	+	+	−	−	
COL3A1	COL3A1	+	+	+	+	+	−	−	
ENG	CD105	+	+	+	+	+	−	−	
HLA-A	HLA-A	+	+	+	+	+	−	−	
ICAM3	CD50	+	+	+	+	+	−	−	
IFNGR1	CD119	+	+	+	+	+	−	−	
IL1R1	CD121a	+	+	+	+	+	−	−	
ITGA1	CD49a	+	+	+	+	+	−	−	
ITGA3	CD49c	+	+	+	+	+	−	−	
ITGA5	CD49e	+	+	+	+	+	−	−	
ITGAE	CD103	+	+	+	+	+	−	−	
ITGAV	CD51	+	+	+	+	+	−	−	
ITGB1	CD29	+	+	−	+	+	−	−	
ITGB2	CD18	+	+	+	−	+	−	−	
NCAM1	CD56	+	+	+	+	+	−	−	
NRP1	CD304	+	+	+	+	+	−	−	
NT5E	CD73	+	+	+	+	+	−	−	
PDGFRA	CD140a	+	+	+	+	+	−	−	
PVRL2	CD112	+	+	+	+	+	−	−	
PVRL3	CD113	+	+	+	+	+	−	−	
RUNX2	RUNX2	+	+	−	−	−	−	−	
SLC3A2	CD98	+	+	+	+	+	−	−	
TFRC	CD71	+	+	+	+	+	−	−	
THY1	CD90	+	+	+	+	+	−	−	
TNFRSF1A	CD120a	+	+	+	+	+	−	−	
VCAM1	CD106	+	+	+	−	+	−	−	
BGLAP	BGLAP	−	−	−	−	−	−	−	II
CD109	CD110	−	−	+	+	+	−	−	
CD34	CD34	−	−	−	−	−	−	−	
CD36	CD36	−	−	−	−	−	−	−	
CD38	CD38	−	−	−	−	−	−	−	
CD4	CD4	−	−	−	−	−	−	−	
CD9	CD9	−	−	+	+	+	−	−	
FLT1	FLT2	−	−	−	−	−	−	−	
HLA-DRB1	HLA-DRB1	−	−	−	−	−	−	−	
HLA-DRB3	HLA-DRB3	−	−	−	−	−	−	−	
HLA-DRB4	HLA-DRB4	−	−	−	−	−	−	−	
HLA-DRB5	HLA-DRB5	−	−	−	−	−	−	−	
HLA-DRB6	HLA-DRB6	−	−	−	−	−	−	−	
IBSP	IBSP	−	−	−	−	−	−	−	
ICAM1	CD54	−	−	−	−	−	−	−	
IL1R2	CD121b	−	−	−	−	−	−	−	
IL2RA	CD25	−	−	−	−	−	−	−	
IL2RB	CD122	−	−	−	−	−	−	−	
IL3RA	CD123	−	−	−	−	−	−	−	
IL4R	CD124	−	−	−	−	−	−	−	
IL6R	CD126	−	−	−	−	−	−	−	
IL7R	CD127	−	−	−	−	−	−	−	
ITGAL	CD11a	−	−	−	−	−	−	−	
ITGB3	CD61	−	−	−	−	+	−	−	
ITGB4	CD104	−	−	−	−	−	−	+	
KDR	CD309	−	−	−	−	−	−	−	
MCAM	CD146	−	−	−	−	−	−	−	
MME CD10	−	−	+	−	−	−	−		
NGFR	CD271	−	−	−	−	−	−	−	
PECAM1	CD31	−	−	−	−	+	−	−	
PROM1	CD133	−	−	−	−	−	−	−	
PTPRCAP	CD45	−	−	−	−	−	−	−	
SELE	CD62e	−	−	−	−	−	−	−	
SELL	CD62l	−	−	−	−	−	−	−	
SELP	CD62p	−	−	−	−	−	−	−	
TNFRSF1B	CD120b	−	−	−	−	+	−	−	
CD164	CD164	+	−	+	+	+	−	−	III
CSF1R	CD115	+	−	−	−	−	−	−	
HLA-DRA	HLA-DRA	+	−	−	−	+	−	−	
ITGA2	CD49b	+	−	+	+	+	−	−	
ITGA4	CD49d	+	−	+	+	−	−	−	
KIT	CD117	+	−	+	+	−	−	−	
SPP1	SPP1	+	−	+	−	−	−	−	
CD14	CD14	−	+	+	+	+	−	−	IV
IV FUT4	CD15	−	+	+	+	+	−	−	
ICAM2	CD102	−	+	+	−	+	−	−	

A distinct set of 601 genes was found to be expressed exclusively in the MSC-TERT cells, amongst these genes are BGLAP, CD115/CSF1R, DLX5 and RUNX2. BGLAP (bone γ-carboxyglutamate (Gla) protein) encodes for osteocalcin, whilst DLX5 and RUNX2 are transcription factors involved in osteoblast differentiation and bone development. The hATSCs-specific gene is of 263, includes the cell surface markers, CD31, CD61 and CD120b, whilst 104 and 111 genes specify the cellular identity of hNSSCs and hASSCs cells respectively. The corresponding signaling and metabolic pathways associated with these cell type-specific gene signatures are presented in supplementary Table 2. The signaling pathways enriched in hMSC-TERT included pathways involved in bone formation e.g. Wnt, TGF-B and MAPK signaling while signaling pathways enriched in hATSCs belonged to adipocyte-relevant metabolic functions e.g. steroid hormone biosynthesis and Linoleic acid metabolism.

## Discussion

In the present study we performed side-by-side comparison of 4 populations of stromal cells derived from adipose tissue, skin and bone marrow. While stromal cell populations can be defined by common set of CD markers, significant differences exist in the growth rate, differentiation potential and molecular signature of these cells.

The bona fide hMSC is derived from bone marrow and generally defined by a set of CD markers and multifunctional differentiation capacity as documented by several studies [19–21]. Our data corroborate the presence of a common set of CD markers expressed in stromal cells from adipose tissue, skin and bone marrow e.g. CD 90, CD73, CD29, CD44, CD105, CD13 and that MSC are negative for hemaptopoietic cell markers: CD45, CD34, CD14, and HLA-DR.

We found differences in the expression of CD146 between stromal cell populations with low levels of expression in adipose tissue MSC compared to skin and bone marrow MSC. CD146 has been identified as a marker for stromal stem cells (MSC) in bone marrow [4]. CD146 defines a population of perivascular and subendothelial cells that is present in different tissues [22]. However, clonal MSC is also present in CD146^-^ bone marrow stromal cell fractions and differences between CD146^+^and CD146^-^ may be related to variation in their functions [23]

We observed significant differences in the growth rates between stromal cells from skin and adipose tissue where hNSSCs exhibited the highest growth rate. These differences may not reflect compartment specific characteristics but most probably reflect differences in donor age: newborn versus adult donors [5]. Alternatively, differences in growth rate may reflect culture heterogeneity with variable proportion of self-renewing versus lineage-committed cells in different stromal cell compartment [24, 25].

Stromal cells from different compartment have been demonstrated in a large number of studies to differentiate into cells in the mesodermal lineages e.g. osteoblasts and adipocytes [6–8, 10–15, 20, 21, 26]. Our results demonstrate that there exist quantitative differences between different stromal cells with respect to their differentiation potential. Bone marrow stromal cells differentiated readily into osteoblastic cells and adipose stromal cells into adipocytes. Skin stromal cells differentiated better to adipocytes than osteoblasts. This suggests the presence of a lineage “imprinting” in different stromal cell compartments that influences the differentiation potential of MSC [27, 28]. Alternatively, we have previously demonstrated that MSC cultures are heterogenous and contains populations of pre-osteoblastic and pre-adipcytic cell populations in bone marrow stromal cultures in addition to the multipotent MSC [24]. The presence of variable number of these committed pre-osteoblastic vs pre-adipocytic cell population may be a factor determining the outcome of in vitro differentiation assays. Further studies of clonal analysis of MSC from different compartment are needed to corroborate this hypothesis.

Molecular profiling based on microarray analysis of steady state gene expression provides insight into the molecular phenotype of the cells and have been used previously in defining the identity of a number of stem cells including MSC and embryonic stem cells [25, 29]. We observed significant differences in the molecular profiling of stromal cells from different compartments, which support the presence of differences in their in vitro growth and differentiation. Interestingly, we found that the 4 stromal cell populations share a common CD marker signature that includes known CD markers of hBM-MSCs. However, this common “public” signature, although it is widely used by different investigators to define the cultured MSC phenotype is not predictive for their in vitro or in vivo behavior [25] and thus cannot be used prospectively to define the nature of the cultured cells [1]. Interestingly, microarray studies revealed the presence of a “private” signature that defines the stromal cells of each compartment and most probably determines their biological behavior. For examples, bone marrow MSC molecular signature was enriched in genes involved in genetic pathways important for bone formation e.g. Wnt and MAPK signaling whereas hATSCs were enriched for genes involved in fatty acid metabolism. Further studies are needed for examining the predictive value of the “private” molecular signature in defining the biological behavior of MSC. The validity of our findings is demonstrated by comparing the molecular phenotype of cell strains with that of primary cells. We have employed hMSC-TERT is an immortalized cell line as a representative model for human bone marrow derived MSC and due to its stable phenotype. Side-by-side comparsion of hMSC-TERT with primary hMSC-STRO + revealed greater similarity between the two cell populations and that they shared 83 % of the expressed genes including similar surface marker phenotype (supplementary figure 6). Similarly, we included two primary fibroblastic cells HFF1 and BJ, which in spite of their fibroblastic nature lacked the expression of the core markers of MSC phenotype.

Our findings have relevance in regenerative medicine. Bone marrow stromal cells have been utilized for their ability for bone tissue replacement and for their immune modulatory effects. However, our findings suggest that stromal cells from other compartments may not be able to replace bone marrow stromal cells in clinical protocols due to the presence of significant differences in their molecular phenotype and differentiation capacity. Thus, the choice of cell source should be based on the aim of clinical application and not on the accessibility of patients’ samples. In this context, the identification of compartment specific MSC molecular signature may help in developing a set of molecular markers that are predictive of the in vivo biological behavior of MSC and that can be used in screening of cultured MSC prior to their clinical use.

## Electronic supplementary material

Below is the link to the electronic supplementary material.

Supplementary Fig. S1 Morphological characteristics of hMSC-TERT, hATSCs, hASSCs and hNSSCs. The human bone marrow stromal (mesenchymal) stem cells (hMSC) immortalized with human telomerase reverse transcriptase gene (hMSC-TERT) and stromal cells isolated from adipose tissue (hATSCs), adult dermal skin (hASSCs) and neonatal foreskin (hNSSCs) cells were cultured using plastic adherence and examined by phase contrast microscopy (bar = 200 μm). (JPEG 347 kb)

High resolution image (TIFF 1488 kb)

Supplementary Fig. S2 Colony forming Unit-fibroblast (CFU-F) formation. Stromal cells isolated from adipose tissue (hATSCs) (a), adult dermal skin (hASSCs) (b) and neonatal foreskin (hNSSCs) (c) were cultured using plastic adherence for 15 days. Colonies were defined as a well-defined group of cells (>40 cells). The number of colonies were determined manually (d). (JPEG 173 kb)

High resolution image (TIFF 3440 kb)

Supplementary Fig. S3 Staining for osteoblast with alkaline phosphatase. The human bone marrow stromal (mesenchymal) stem cells (hMSC) immortalized with human telomerase reverse transcriptase gene (hMSC-TERT) and stromal cells isolated from adipose tissue (hATSCs), adult dermal skin (hASSCs) and neonatal foreskin (hNSSCs) cells were cultured using plastic adherence and in the presence of osteoblast differentiation medium (OB differentiated) or under control conditions (control). Osteoblastic cells were visualized by positive staining for alkaline phosphatase (bar = 200 μm). (JPEG 74 kb)

High resolution image (TIFF 2704 kb)

Supplementary Fig. S4 Staining for adipocytes with Oil red O. The human bone marrow stromal (mesenchymal) stem cells (hMSC) immortalized with human telomerase reverse transcriptase gene (hMSC-TERT) and stromal cells isolated from adipose tissue (hATSCs), adult dermal skin (hASSCs) and neonatal foreskin (hNSSCs) cells were cultured using plastic adherence and in the presence of adipocyte induction medium (AD differentiated) or under control conditions (control). Adipocyte differentiated was demonstrated by staining with Oil red O (bar = 200 μm). (JPEG 99 kb)

High resolution image (TIFF 2895 kb)

Supplementary Fig. S5 Microarray-based analysis of hMSC-TERT, hATSCs, hASSCs and hNSSCs. The human bone marrow stromal (mesenchymal) stem cells (hMSC) immortalized with human telomerase reverse transcriptase gene (hMSC-TERT) and stromal cells isolated from adipose tissue (hATSCs), adult dermal skin (hASSCs) and neonatal foreskin (hNSSCs) cells were cultured through plastic adherence. RNA was isolated from (hMSC-TERT) and stromal cells derived from adipose tissue (hATSCs), adult dermal skin (hASSCs) and neonatal foreskin (hNSSCs) cells were subjected to microarray analysis. Additionally, we included normal primary bone marrow hMSC isolated by Stro-1 antibody immune magenetic panning (hMSC-STRO basal) and two commercial fibroblastic cell lines: neonatal foreskin fibroblasts BJ and HFF1. (A, B) Hierarchical clustering of the distinct cell populations. (C) Table showing correlation co-efficients-R^2^ between the different cell populations. (JPEG 158 kb)

Supplementary Fig. S6 Transcriptional similarity between hMSC-TERT and hMSC-STRO^+^ basal. The molecular phenotype of the human bone marrow stromal (mesenchymal) stem cells (hMSC) immortalized with human telomerase reverse transcriptase gene (hMSC-TERT) was compared to normal primary bone marrow hMSC isolated by Stro-1 antibody (hMSC-STRO). Venn diagram showing the distinct and overlapping gene signatures of hMSC-TERT and hMSC-STRO. The majority of transcripts were common to both cell types (6895 corresponding to 83 % of the total). (JPEG 29 kb)

Supplementary Table S1 Mean levels of stromal cells-associated, hematopoietic and endothelial markers expression on different cell population. (DOC 38 kb)

Supplementary Table S2 Spreadsheet 1: List of genes specific for hMSC-TERT. Spreadsheet 2: List of genes specific for hNSSCs. Spreadsheet 3: List of genes specific for hASSCs. Spreadsheet 4: List of genes specific for hATSCs. Spreadsheet 5: List of genes in common between hMSC-TERT and hNSSCs. Spreadsheet 6: List of genes in common between hMSC-TERT and hASSCs. Spreadsheet 7: List of genes in common between hMSC-TERT and hATSCs. Spreadsheet 8: List of genes in common between all samples expect for hATSCs. Spreadsheet 9: List of genes in common between all samples (XLS 397 kb)
