# Supplementary figures and images for: Human Stromal (Mesenchymal) Stem Cells from Bone Marrow, Adipose Tissue and Skin Exhibit Differences in Molecular Phenotype and Differentiation Potential
Source: Stem Cell Rev Rep. 2012 Apr 14;9(1):32–43. doi: 10.1007/s12015-012-9365-8 (PMC3563956; doi:10.1007/s12015-012-9365-8)

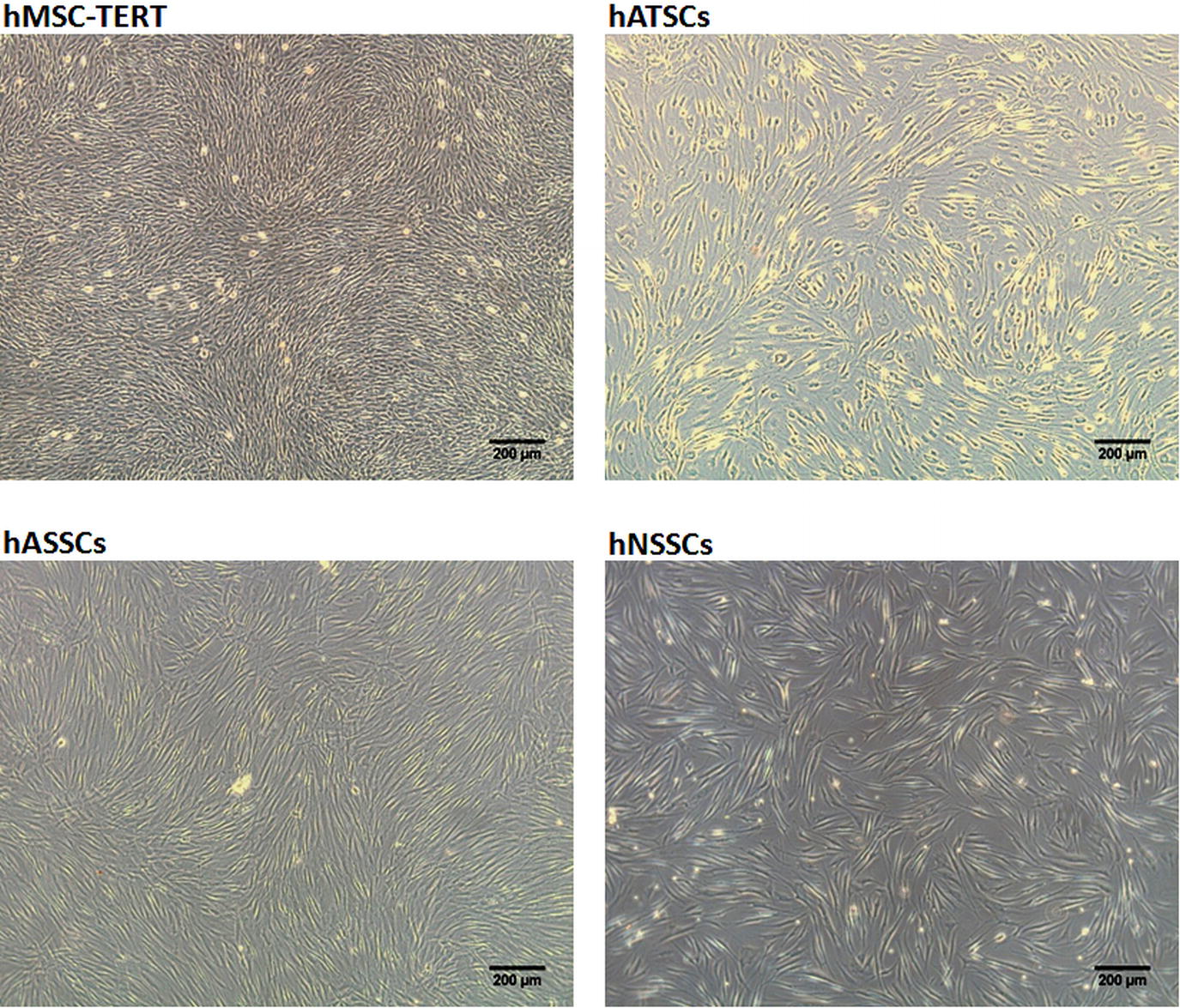

Supplement: Supplementary file 1 — Morphological characteristics of hMSC-TERT, hATSCs, hASSCs and hNSSCs. The human bone marrow stromal (mesenchymal) stem cells (hMSC) immortalized with human telomerase reverse transcriptase gene (hMSC-TERT) and stromal cells isolated from adipose tissue (hATSCs), adult dermal skin (hASSCs) and neonatal foreskin (hNSSCs) cells were cultured using plastic adherence and examined by phase contrast microscopy (bar = 200 μm). (JPEG 347 kb) [file 12015_2012_9365_Fig5_HTML.jpg]

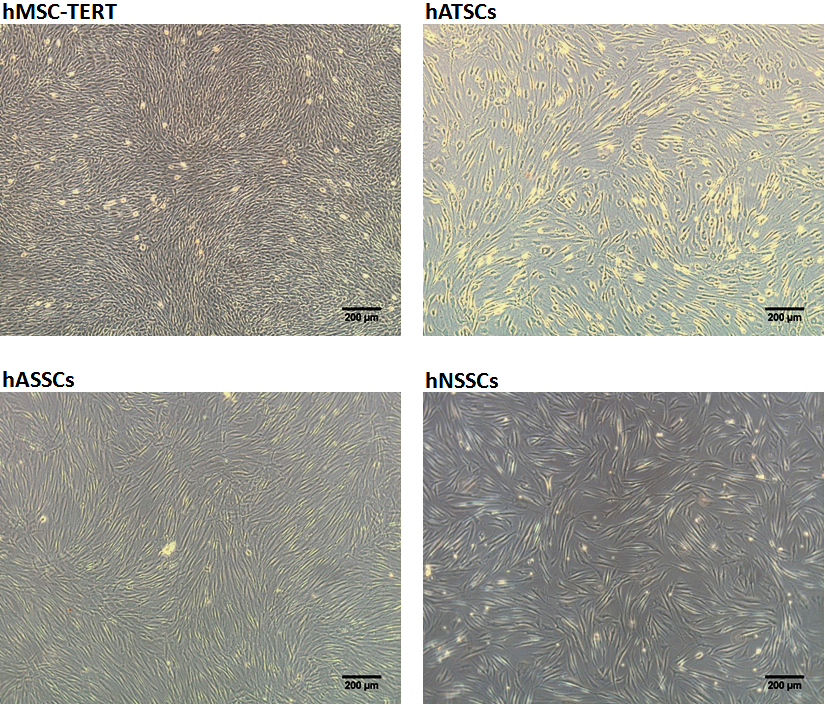

Supplement: Supplementary file 2 — High resolution image (TIFF 1488 kb) [file 12015_2012_9365_MOESM1_ESM.tiff]

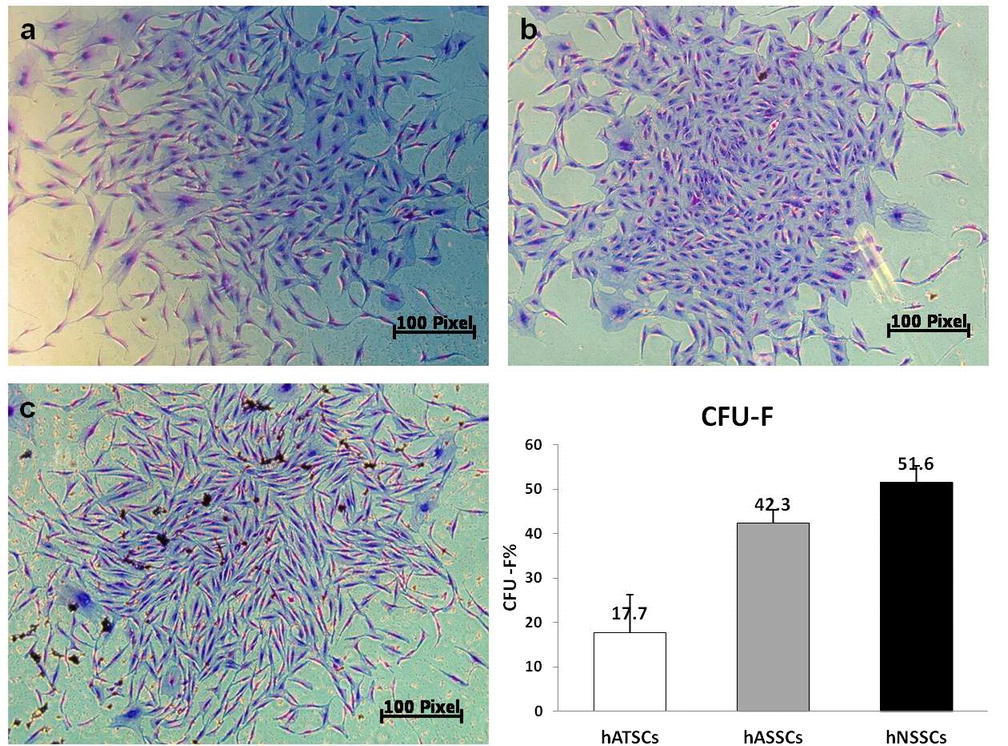

Supplement: Supplementary file 3 — Colony forming Unit-fibroblast (CFU-F) formation. Stromal cells isolated from adipose tissue (hATSCs) (a), adult dermal skin (hASSCs) (b) and neonatal foreskin (hNSSCs) (c) were cultured using plastic adherence for 15 days. Colonies were defined as a well-defined group of cells (>40 cells). The number of colonies were determined manually (d). (JPEG 173 kb) [file 12015_2012_9365_Fig6_HTML.jpg]

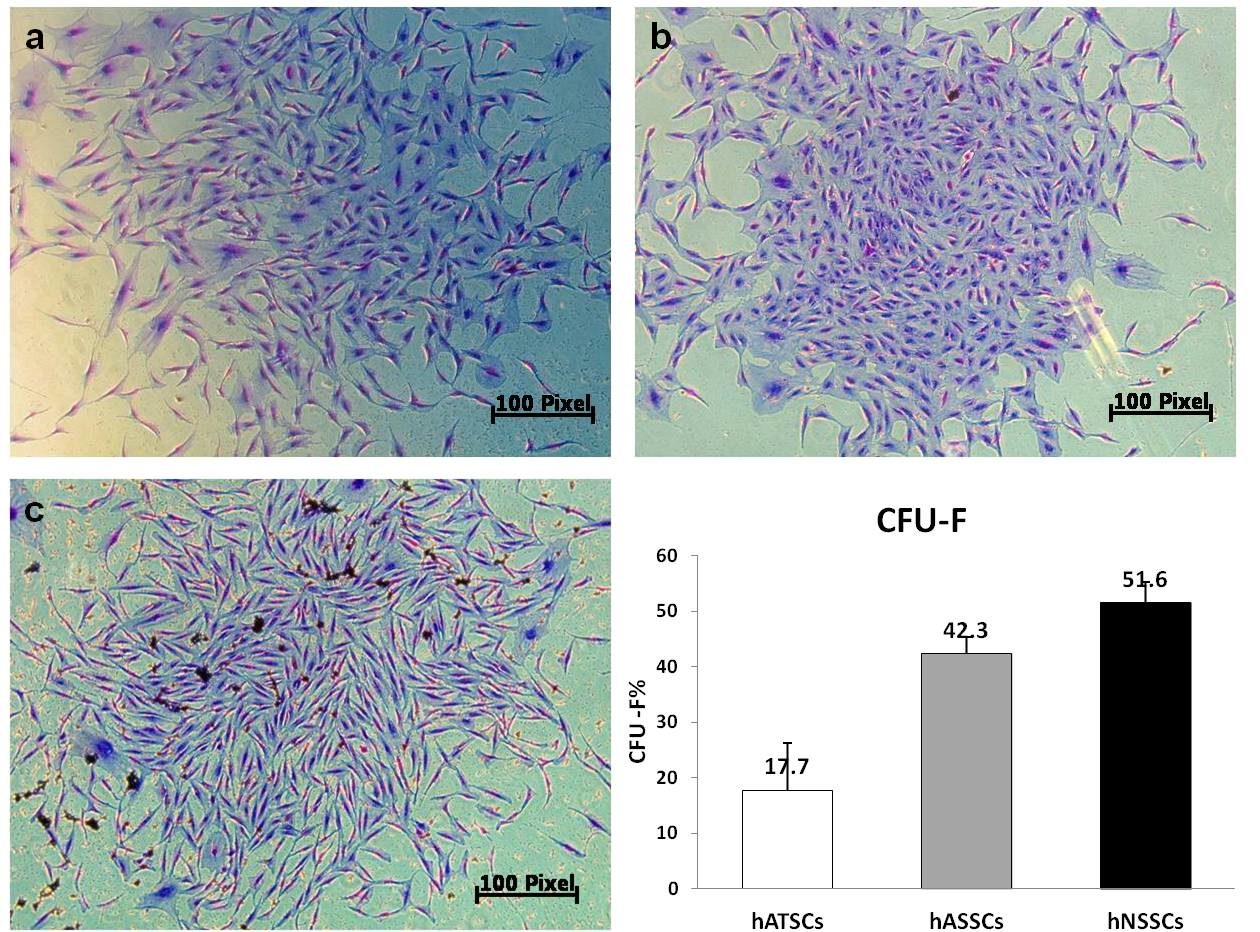

Supplement: Supplementary file 4 — High resolution image (TIFF 3440 kb) [file 12015_2012_9365_MOESM2_ESM.tiff]

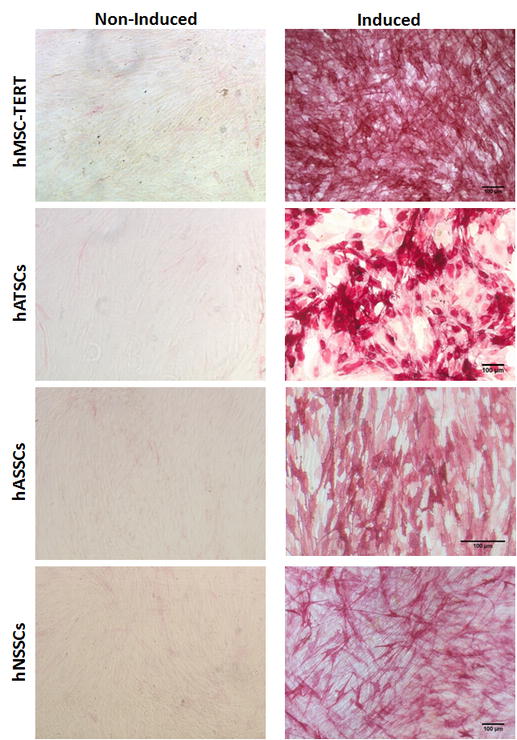

Supplement: Supplementary file 5 — Staining for osteoblast with alkaline phosphatase. The human bone marrow stromal (mesenchymal) stem cells (hMSC) immortalized with human telomerase reverse transcriptase gene (hMSC-TERT) and stromal cells isolated from adipose tissue (hATSCs), adult dermal skin (hASSCs) and neonatal foreskin (hNSSCs) cells were cultured using plastic adherence and in the presence of osteoblast differentiation medium (OB differentiated) or under control conditions (control). Osteoblastic cells were visualized by positive staining for alkaline phosphatase (bar = 200 μm). (JPEG 74 kb) [file 12015_2012_9365_Fig7_HTML.jpg]

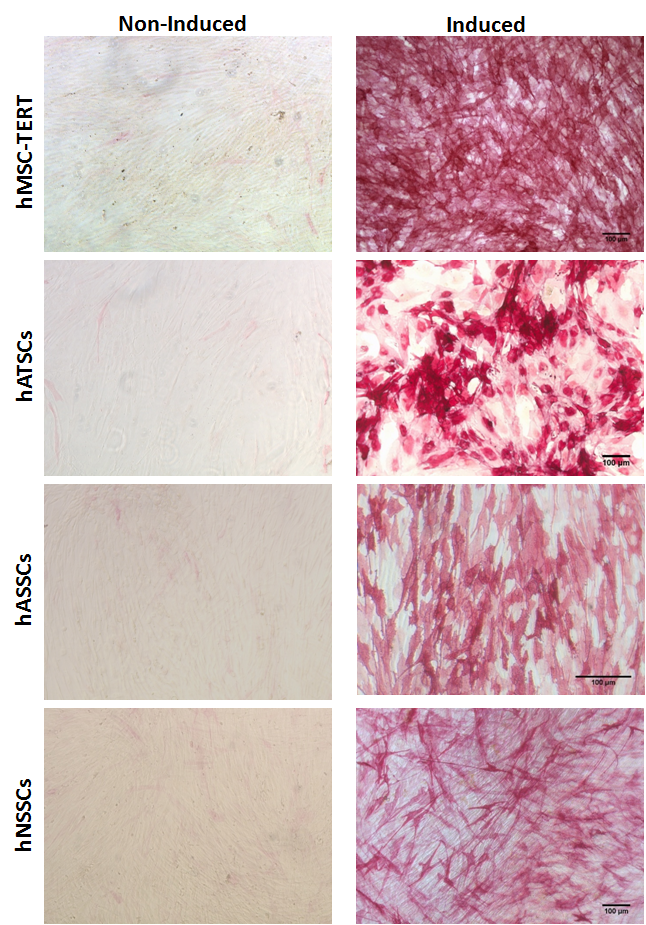

Supplement: Supplementary file 6 — High resolution image (TIFF 2704 kb) [file 12015_2012_9365_MOESM3_ESM.tiff]

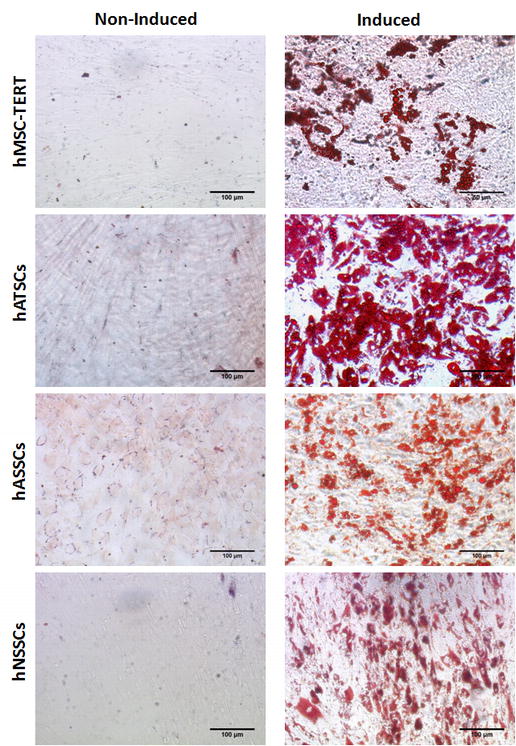

Supplement: Supplementary file 7 — Staining for adipocytes with Oil red O. The human bone marrow stromal (mesenchymal) stem cells (hMSC) immortalized with human telomerase reverse transcriptase gene (hMSC-TERT) and stromal cells isolated from adipose tissue (hATSCs), adult dermal skin (hASSCs) and neonatal foreskin (hNSSCs) cells were cultured using plastic adherence and in the presence of adipocyte induction medium (AD differentiated) or under control conditions (control). Adipocyte differentiated was demonstrated by staining with Oil red O (bar = 200 μm). (JPEG 99 kb) [file 12015_2012_9365_Fig8_HTML.jpg]

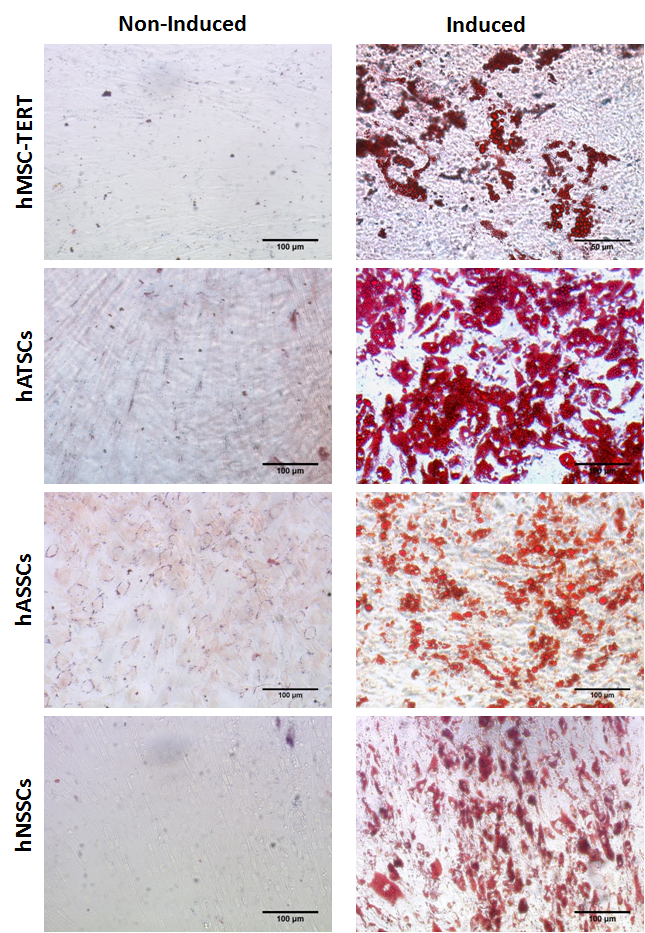

Supplement: Supplementary file 8 — High resolution image (TIFF 2895 kb) [file 12015_2012_9365_MOESM4_ESM.tiff]

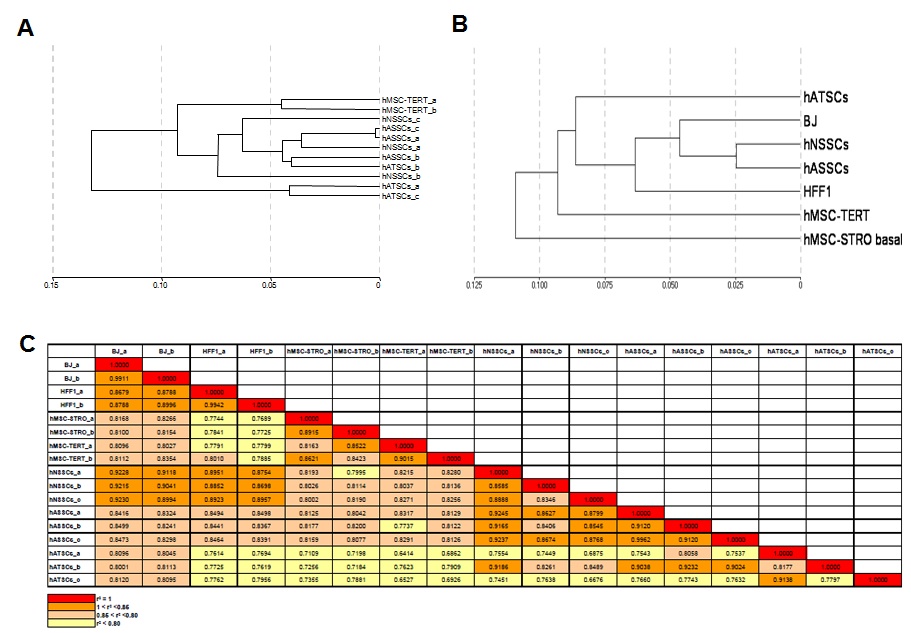

Supplement: Supplementary file 9 — Microarray-based analysis of hMSC-TERT, hATSCs, hASSCs and hNSSCs. The human bone marrow stromal (mesenchymal) stem cells (hMSC) immortalized with human telomerase reverse transcriptase gene (hMSC-TERT) and stromal cells isolated from adipose tissue (hATSCs), adult dermal skin (hASSCs) and neonatal foreskin (hNSSCs) cells were cultured through plastic adherence. RNA was isolated from (hMSC-TERT) and stromal cells derived from adipose tissue (hATSCs), adult dermal skin (hASSCs) and neonatal foreskin (hNSSCs) cells were subjected to microarray analysis. Additionally, we included normal primary bone marrow hMSC isolated by Stro-1 antibody immune magenetic panning (hMSC-STRO basal) and two commercial fibroblastic cell lines: neonatal foreskin fibroblasts BJ and HFF1. (A, B) Hierarchical clustering of the distinct cell populations. (C) Table showing correlation co-efficients-R2 between the different cell populations. (JPEG 158 kb) [file 12015_2012_9365_MOESM5_ESM.jpg]

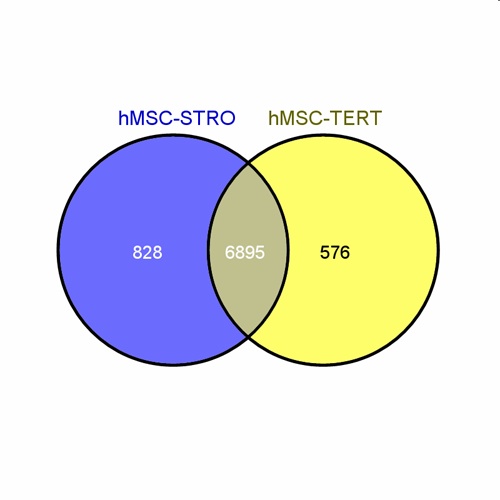

Supplement: Supplementary file 10 — Transcriptional similarity between hMSC-TERT and hMSC-STRO+ basal. The molecular phenotype of the human bone marrow stromal (mesenchymal) stem cells (hMSC) immortalized with human telomerase reverse transcriptase gene (hMSC-TERT) was compared to normal primary bone marrow hMSC isolated by Stro-1 antibody (hMSC-STRO). Venn diagram showing the distinct and overlapping gene signatures of hMSC-TERT and hMSC-STRO. The majority of transcripts were common to both cell types (6895 corresponding to 83 % of the total). (JPEG 29 kb) [file 12015_2012_9365_MOESM6_ESM.jpg]
