# Supplementary material for: Human Stromal (Mesenchymal) Stem Cells from Bone Marrow, Adipose Tissue and Skin Exhibit Differences in Molecular Phenotype and Differentiation Potential
Source: Stem Cell Rev Rep. 2012 Apr 14;9(1):32–43. doi: 10.1007/s12015-012-9365-8 (PMC3563956; doi:10.1007/s12015-012-9365-8)
Supplement: Supplementary file 11 — Mean levels of stromal cells-associated, hematopoietic and endothelial markers expression on different cell population. (DOC 38 kb) [file 12015_2012_9365_MOESM7_ESM.doc]

|  | **Lymphocytes** | **Monocytes** | **hMSC-TERT** | **hATSCs** | **hASSCs** | **hNSSCs** |
| --- | --- | --- | --- | --- | --- | --- |
| **CD31** | 41±0.11 | 99±0.01 | 1±0.004 | 1±0.01 | 1±0.001 | 1±0.001 |
| **HLADR** | 13±0.02 | 83±0.11 | 16±0.28 | 1±0.01 | 1±0.001 | 1±0.001 |
| **CD34** | 1±0.003 | 1±0.01 | 1±0.003 | 5±0.1 | 1±0.001 | 1±0.001 |
| **CD45** | 98±0.01 | 98±0.002 | 1±0.001 | 1±0.01 | 1±0.002 | 1±0.0002 |
| **CD14** | 1±0.01 | 93±0.02 | 1±0.002 | 1±0.01 | 1±0.002 | 1±0.003 |
| **CD44** | 100±0.003 | 100±0.003 | 100±0.0005 | 100±0.002 a† | 100±0.0003 b† | 100±0.001 b† |
| **CD146** | 1±0.005 | 1±0.002 | 58±0.34 | 5±0.1 a† | 65±0.30 b* | 65±0.32 b* |
| **CD105** | 9±0.05 | 9±0.1 | 100±0.005 | 100±0.01 | 100±0.004 | 99±0.01 |
| **CD29** | 93±0.1 | 93±0.002 | 100±0.0004 | 98±0.02 a‡ | 100±0 b‡ | 100±0.004 |
| **CD13** | 4±0.01 | 4±0.2 | 100±0.004 | 98±0.04 | 100±0.001 | 100±0.004 |
| **CD90** | 17±0.2 | 18±0.2 | 97±0.045 | 100±0.003 a‡ | 100±0.0004 a‡ | 100±0.0004 a‡ |
| **CD73** | 18±0.02 | 18±0.01 | 100±0.001 | 99±0.01 a* | 100±0.001 b* | 100±0.0004 b* |

**Supplementary Table 1**

**Mean levels of stromal cells-associated, hematopoietic and endothelial markers expression on different cell population**

Values are expressed as mean% ± SD in each group TERT 20 - positive control, lymphocytes and monocytes from peripheral blood used as reagent control. Statistical Analyses (* , † and ‡ represent P< 0.001, P< 0.01 and P< 0.05) Group 1 Vs Group 2 and Group 3. (*n*=6)
